# 

**Published:** 1905-02

**Authors:** 


Twentieth Year.

Vol. XX. FEBRUARY, 1905. No. 8.

### TEXAS
### Medical Journal.

Subscription, $1.00 a Year, in Advance.

Single Copies, 10 Cents.

PUBLISHED AT AUSTIN, TEXAS, BY

F. E. DANIEL, M. D.,

Proprietor.

PRINTED BY THE VON BOECKMANN-JONES CO.

Entered at the Postoffice at Austin, Texas, as Second-class Mail Matter.

